# The evolving concept of conversion surgery for upfront unresectable upper gastrointestinal and hepato-pancreato-biliary cancers: comprehensive review

**DOI:** 10.1093/bjsopen/zraf070

**Published:** 2025-07-09

**Authors:** Giampaolo Perri, Jennie Engstrand, Robin D Wright, Sebastiaan F C Bronzwaer, Tiuri E Kroese, Biying Huang, Belkacem Acidi, Alessandro Vitale, Hop S Tran Cao, Richard van Hillegersberg, Magnus Nilsson, Ernesto Sparrelid, Matthew H G Katz, Giovanni Marchegiani, Umberto Cillo

**Affiliations:** Hepato-pancreato-biliary and Liver Transplant Surgery Unit, Department of Surgical, Oncological and Gastroenterological Sciences (DiSCOG), University of Padua, Padua, Italy; Department of General Surgery, IRCCS Azienda Ospedaliero-Universitaria di Bologna, Maggiore Hospital, Bologna, Italy; Division of Surgery and Oncology, Department of Clinical Science, Intervention and Technology, Karolinska Institutet, Karolinska University Hospital, Stockholm, Sweden; Department of Surgical Oncology, The University of Texas MD Anderson Cancer Center, Houston, Texas, USA; Department of Surgery, University Medical Center Utrecht, Utrecht University, Utrecht, the Netherlands; Department of Radiation Oncology, University Hospital Zurich, University of Zurich, Zurich, Switzerland; Division of Surgery and Oncology, Department of Clinical Science, Intervention and Technology, Karolinska Institutet, Karolinska University Hospital, Stockholm, Sweden; Department of Surgical Oncology, The University of Texas MD Anderson Cancer Center, Houston, Texas, USA; Hepato-pancreato-biliary and Liver Transplant Surgery Unit, Department of Surgical, Oncological and Gastroenterological Sciences (DiSCOG), University of Padua, Padua, Italy; Department of Surgical Oncology, The University of Texas MD Anderson Cancer Center, Houston, Texas, USA; Department of Surgery, University Medical Center Utrecht, Utrecht University, Utrecht, the Netherlands; Division of Surgery and Oncology, Department of Clinical Science, Intervention and Technology, Karolinska Institutet, Karolinska University Hospital, Stockholm, Sweden; Division of Surgery and Oncology, Department of Clinical Science, Intervention and Technology, Karolinska Institutet, Karolinska University Hospital, Stockholm, Sweden; Department of Surgical Oncology, The University of Texas MD Anderson Cancer Center, Houston, Texas, USA; Hepato-pancreato-biliary and Liver Transplant Surgery Unit, Department of Surgical, Oncological and Gastroenterological Sciences (DiSCOG), University of Padua, Padua, Italy; Hepato-pancreato-biliary and Liver Transplant Surgery Unit, Department of Surgical, Oncological and Gastroenterological Sciences (DiSCOG), University of Padua, Padua, Italy

## Abstract

**Background:**

In the absence of a commonly accepted definition, conversion surgery is generally considered as surgical resection with the intent of prolonging survival after non-surgical induction therapy in patients with upfront unresectable disease at diagnosis. Despite the heterogeneity of possible targets, conversion surgery is a quickly evolving concept, with commonalities for upper gastrointestinal (UGI) and hepato-pancreato-biliary (HPB) malignancies.

**Methods:**

A comprehensive narrative review of the most recent and relevant literature was conducted by experts in the field of different UGI and HPB tumours.

**Results:**

The increased interest of the surgical scientific community in the concept of conversion surgery can be explained by the continuous improvements in non-surgical therapies aimed at controlling the systemic tumour burden and the local extension of cancer, supported by improvements in surgical outcomes for advanced resections in expert centres. The toolbox of the surgical oncologist seeking conversion in the case of unresectable UGI and HBP tumours is large and includes (but is not limited to) systemic chemotherapy, (chemo)radiation, targeted therapy/immunotherapy, locoregional ablation techniques, intra-arterial therapies, liver hypertrophy induction techniques, treatments of underlying medical conditions, and prehabilitation.

**Conclusions:**

Conversion surgery represents a powerful instrument to prolong the survival of patients with unresectable UGI and HPB malignancies. However, most of the available evidence is of a low level and at very high risk of selection bias. Alongside a profound understanding of (and respect for) the biology of cancer, which remains key to selecting appropriate patients and avoiding non-therapeutic surgeries, a commonly accepted definition is urgently needed to standardize practice, monitor outcomes, and improve the quality of research.

## Teaser

Despite the heterogeneity of targets and in the absence of a commonly accepted definition, conversion surgery for upper gastrointestinal and hepato-pancreato-biliary malignancies is an evolving concept, generally intended as surgical resection with curative intent after non-surgical induction therapy, in patients with upfront unresectable disease at diagnosis.

## Introduction

Despite the absence of a commonly accepted definition, the concept of conversion surgery for upper gastrointestinal (UGI) and hepato-pancreato-biliary (HPB) cancers is rapidly evolving. This is primarily due to the higher efficacy of systemic therapies, shifting paradigms of treatment sequencing for surgical oncology, the increased availability of different locoregional treatments, and improvements in surgical outcomes for extended resections. Despite nuances related to differences in biology and anatomy, conversion surgery for UGI and HPB malignancies can be generally defined as surgical resection with curative intent after non-surgical induction therapy following a favourable response in patients with upfront unresectable disease at diagnosis in whom surgery alone would fail to provide curative resection and/or improve overall survival.

Unresectability is also an evolving and heterogeneous concept in UGI and HPB surgical oncology, and can be defined on the basis of either technical aspects, such as local tumour invasion/burden and the need to preserve essential organ function, or biological aspects, such as a tumour's aggressiveness, the degree of systemic spread, and a patient's frailty and/or co-morbidities.

However, many of the factors determining an increased risk of non-therapeutic surgery are actionable, and achieving a successful reduction in this risk is a prerequisite for conversion surgery, as well as modern oncological surgery, which ultimately aims to prolong overall survival. The term ‘conversion’ refers to the effect of preoperative therapies on a tumour, not only with regard to its anatomical constraints but in particular to its biology, allowing for better patient selection and shifting from anatomical-based resectability towards prognosis-based resectability.

**Fig. 1:**
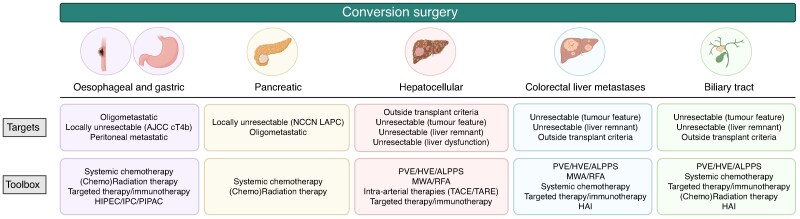
Specific targets of conversion surgery and the surgeon oncologist's toolbox, among different UGI and HPB malignancies AJCC, American Joint Committee on Cancer; LAPC, locally advanced pancreatic cancer; NCCN, National Comprehensive Cancer Network; HIPEC, hyperthermic intraperitoneal chemotherapy; IPC, intraperitoneal catheter-based chemotherapy; PIPAC, pressurized intraperitoneal aerosol chemotherapy; MWA, microwave ablation; RFA, radiofrequency ablation; TACE, transarterial chemoembolization; TARE, transarterial radioembolization; PVE, portal vein embolization; HVE, hepatic vein embolization; ALPPS, associating liver partition and portal vein ligation for staged hepatectomy; HAI, hepatic arterial infusion chemotherapy. (Created with BioRender.com.)

The toolbox of the surgical oncologist seeking conversion in unresectable UGI and HBP tumours is large and includes (but is not limited to) systemic chemotherapy, (chemo)radiation, targeted therapy/immunotherapy, locoregional ablation techniques, intra-arterial therapies, liver hypertrophy induction techniques, treatment of underlying medical conditions, and prehabilitation (*Fig. 1*). In the absence of a consensus defining both conversion surgery and its techniques in UGI and HPB surgical oncology, this narrative review aims to provide a comprehensive overview of the existing literature on the topic for the most common UGI (oesophageal and gastric cancer) and HPB (pancreatic cancer, hepatocellular carcinoma (HCC), colorectal liver metastases, and biliary tract cancer) malignancies.

## Methods

A comprehensive narrative review was conducted by experts in the field of conversion surgery for various UGI and HPB tumours. The most relevant and recent literature available for each specific area was discussed; current scientific gaps were highlighted, and future perspectives were also discussed. The review was divided into sections addressing specific tumours, with the structure of each section left to the experts’ preference. This approach allowed for a free discussion of the most important topics, respecting the specific peculiarities of each disease. In the absence of a commonly accepted definition, the interpretation of the term ‘conversion surgery’ in each context was left up to the experts.

## Oesophageal cancer

This section aims to provide an overview of the concept of conversion surgery specifically for induced oligometastatic disease (OMD) in oesophageal cancer. Conversion surgery could also be applied for locally unresectable (cT4b) oesophageal cancer or peritoneal metastases.

### Locally unresectable cT4b disease

Treatment for cT4b oesophageal cancer may consist of definitive chemoradiotherapy followed by salvage esophagectomy^1,2^. Definitive chemoradiotherapy usually consists of radiotherapy (RT) up to 50.4 Gy, as opposed to 41.4 Gy used in a neoadjuvant setting with concomitant carboplatin and paclitaxel chemotherapy^3,4^. A prospective phase II trial on 48 patients undergoing conversion surgery after induction chemo(radio)therapy demonstrated improved overall survival rates for patients who achieved R0 resection (19 patients)^5^. In that trial, the 3-year overall survival rates were 71.4% for all patients who underwent R0 resection, and 30.1% for non-R0 patients^5^. In addition, a retrospective cohort study on salvage robot-assisted minimally invasive oesophagectomy after definitive chemoradiotherapy demonstrated higher 12-month overall survival rates in patients who underwent salvage robotic oesophagectomy compared with those who did not undergo surgery (83 *versus* 29%, respectively)^1^. Moreover, nearly all patients achieved R0 resection (22 of 24 patients, 91.7%), and the 24-month disease-free-survival rate was 68% among patients who achieved an R0 resection after robot-assisted minimally invasive oesophagectomy^1^. A pathological complete response was achieved in 54% of resected patients^1^.

### Peritoneal metastases

Peritoneal metastatic disease represents a distinct biological subset of metastatic disease confined to the peritoneal cavity^6,7^. Metastatic disease from oesophageal cancer most frequently occurs in the extraregional lymph nodes (53%) and liver (49%), with the peritoneum being the fifth most frequent metastatic site (11%)^8^. Peritoneal metastases are more commonly associated with gastric cancer than oesophageal cancer. Therefore, peritoneal metastases are discussed in greater detail in the gastric cancer section. Currently, no trials have investigated the role of local treatment of peritoneal metastases (for example, cytoreductive surgery plus hyperthermic intraperitoneal chemotherapy (HIPEC)) for oesophageal cancer.

#### Induced *versus* genuine OMD

**Fig. 2:**
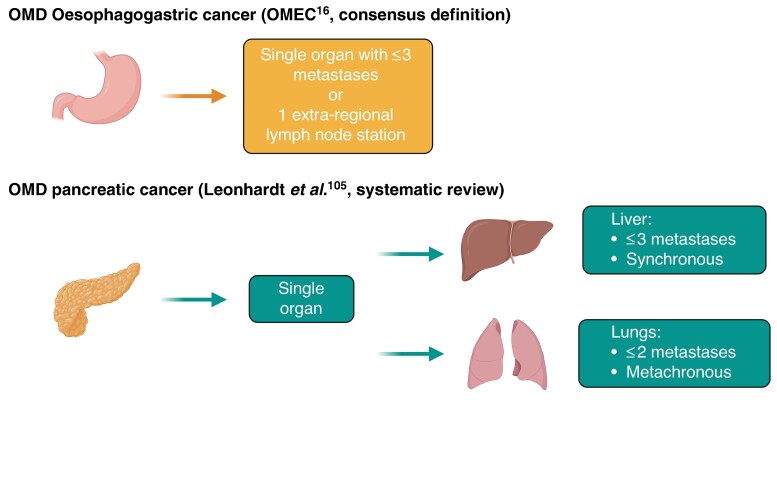
Definitions of oligometastatic disease in oesophagogastric and pancreatic cancers OMD, oligometastatic disease; OMEC, OligoMetastatic Esophagogastric Cancer project. (Created with BioRender.com.)

**Fig. 3:**
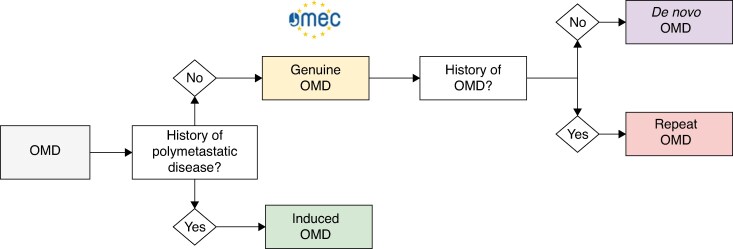
Overview of different types of oesophageal oligometastatic disease Patients have induced OMD in case of a history of polymetastatic disease before diagnosis of OMD. Patients with genuine OMD have no history of polymetastatic disease before diagnosis of OMD. In the OMEC project, only patients with genuine OMD were included. OMD, oligometastatic disease; OMEC, OligoMetastatic Esophagogastric Cancer project.

OMD is defined as a disease state that is situated between localized disease and widespread distant metastatic disease (for example, peritoneal or pleural metastases)^9,10^ (*Fig. 2*). Kroese *et al.*^11^ estimated that approximately 25% of patients with metastatic oesophageal cancer present with OMD. In that study, OMD was defined as five or fewer distant metastases, excluding peritoneal or pleural metastases^11^. The concept of OMD implies that local metastasis-directed treatment in addition to systemic therapy could improve oncological outcomes^12,13^. In the European Organisation for Research and Treatment of Cancer (EORTC) classification system^10^, induced OMD is a subtype of OMD characterized by patients who have polymetastatic disease initially that becomes oligometastatic after induction chemo(radio)therapy^10^. The counterpart of induced OMD in the EORTC classification system is genuine OMD (that is, OMD at first presentation without a history of polymetastatic disease)^10^. The two subtypes that fall under genuine OMD include *de novo* OMD (that is, first-time diagnosis of OMD without a previous history of polymetastatic disease) and repeat OMD (that is, a history of OMD before the current diagnosis of OMD). *De novo* OMD can be subdivided into synchronous OMD (*de novo* OMD diagnosed within 6 months of diagnosis of the primary tumour) or metachronous OMD (*de novo* OMD diagnosed >6 months after the primary tumour)^10^. An overview of the different subtypes of OMD is shown in *Fig. 3*.

Until recently, a uniform definition of OMD in oesophageal cancer was lacking, leading to different interpretations in ongoing studies. For example, the ongoing phase III RENAISSANCE trial^14^, which is investigating the survival effect of adding surgery to treatment of genuine OMD, has defined OMD as the first-time diagnosis of retroperitoneal lymph node metastases only and/or a maximum of one incurable organ site (including peritoneal metastases) that is potentially resectable or locally controllable with or without retroperitoneal lymph nodes (that is, genuine OMD). In the RENAISSANCE trial^14^ , patients with genuine OMD receive four cycles of 5-fluorouracil, leucovorin, oxaliplatin, and docetaxel (FLOT) chemotherapy alone or with trastuzumab if they are positive for human epidermal growth factor receptor 2 (HER2). Patients without disease progression after four cycles are randomized 1 : 1 to receive additional chemotherapy cycles or surgical resection of primary and metastases, followed by subsequent chemotherapy. However, the ongoing phase III trial (NCT04248452) by the Eastern Cooperative Oncology Group (ECOG) defined OMD in oesophagogastric adenocarcinoma as the first-time diagnosis of three or fewer metastatic lesions in addition to the primary tumour (that is, *de novo* OMD). This disparity in definitions prompted the establishment of the European multidisciplinary OligoMetastatic Esophagogastric Cancer (OMEC) project^15^. The aim of the pan-European initiative is to develop a standardized definition of OMD in patients with oesophagogastric cancer. In a step-wise approach, the OMEC project achieved a consensus across 16 European countries and 49 expert centres stating that genuine OMD in patients with oesophagogastric cancer is limited to the involvement of a single organ with three or fewer metastases or one extraregional lymph node station^16^ (*Fig. 2*). In addition, subdivisions were made depending on the organ or lymphatic site. In the OMEC project, recommendations regarding the definition and treatment of OMD in oesophagogastric cancer primarily focused on genuine OMD. A consensus was reached that patients with OMD would first start with induction systemic therapy for at least 3 months, followed by restaging^16^. Local treatment could be considered at restaging in patients with OMD without progression in the number of metastases, whereas progression in size was allowed at the metastatic lesion(s) only^16^. No specific recommendations for induced OMD were made.

#### Patient selection

Currently, the most relevant diagnostic method for defining OMD is imaging^10,17^. ^18^F-Fluorodeoxyglucose positron emission tomography/computed tomography (^18^F-FDG PET/CT) has been shown to improve the detection of metastases in patients with oesophageal cancer compared with CT alone^17–19^. Accordingly, ^18^F-FDG PET/CT was recommended in the OMEC project^15^ for baseline imaging to distinguish genuine OMD from polymetastatic disease, and for restaging after induction systemic therapy to select patients for local treatment of OMD. However, a challenging aspect lies in determining which lesions to target for treatment, particularly considering that patients with induced OMD have vanishing metastatic lesions on imaging (as per the definition). In addition, it is crucial to recognize the inherent limitations of imaging modalities, including the potential for false positives and false negatives. Therefore, upon restaging after induction therapy, uncertainty persists regarding whether previously identified lesions have genuinely disappeared or truly remain persistent. In addition, from an ethical standpoint, it could be challenging to obtain informed consent from the patient for an invasive procedure carrying the risk of morbidity and potential mortality, especially when the benefit of treating vanishing lesions remains uncertain.

A novel approach to patient selection for local treatment of induced OMD could be the use of circulating tumour DNA (ctDNA)^20^, which refers to fragments of DNA originating from tumour cells circulating in the bloodstream alongside cell-free DNA. Clearance of ctDNA following systemic therapy serves as a marker for improved outcomes, potentially offering a way of identifying candidates for treatment of induced OMD. Accordingly, a phase II study (NCT04931420) is currently investigating the role of local treatment for genuine OMD in addition to standard-of-care (SOC) chemotherapy *versus* SOC chemotherapy alone in patients with adenocarcinoma of the oesophagus, stomach, or pancreas with ctDNA clearance after chemotherapy^21^. Patients with genuine OMD in that trial will first undergo induction systemic therapy, and patients without progression after induction chemotherapy and having undetectable ctDNA will then be randomized (1 : 1) to either sequential surgery of the primary tumour and all metastases followed by maintenance chemotherapy or continued chemotherapy alone. The primary endpoint is progression-free survival, and a total of 48 patients will be included.

Selecting patients for conversion surgery for induced OMD is challenging, because the role of local treatment in OMD in oesophageal cancer is not yet definitively established. Current literature regarding induced OMD is limited, and there is a noticeable lack of both prospective and retrospective studies in this area. Consequently, in the absence of more comprehensive evidence on induced OMD, data on genuine OMD in oesophagogastric cancer or induced OMD in other types of cancer could be analysed.

#### Published studies on genuine OMD in oesophageal cancer

To date, there have been three Chinese prospective trials^13,22,23^ (including one randomized controlled trial) in patients with genuine OMD in oesophageal squamous cell cancer. These three prospective trials have (mainly) evaluated the value of stereotactic body radiation therapy (SBRT) for the treatment of genuine OMD. In addition, there has been one German prospective trial^12^ in patients with genuine OMD in gastroesophageal junction adenocarcinoma.

The randomized controlled ESO-Shanghai 13 phase II trial^22^ showed improved progression-free and overall survival with combined local treatment and systemic therapy *versus* systemic therapy alone in patients with genuine oligometastatic squamous cell carcinoma of the oesophagus (stratified hazard ratio (HR) 0.26 (95% confidence interval (c.i.) 0.16 to 0.42) and 0.42 (95% c.i. 0.24 to 0.74), respectively). Most patients in the ESO-Shanghai 13 trial^22^ had one (43%) or two lesions (42%) located in one organ (79%) or two organs (21%). The OMD was located in the cervical lymph nodes, lung, abdominal lymph nodes, or liver. Local treatment for OMD was RT (83%) or surgery (8%) or thermal ablation (4%), and local treatment for the primary tumour was chemoradiotherapy (64%) or oesophagectomy (36%)^22^. Treatment in the ESO-Shanghai 13 trial commenced within 2 weeks of randomization. In both groups, four cycles of SOC chemotherapy were delivered according to the initial protocol. Acceptable options for chemotherapy were paclitaxel and cisplatin for first-line systemic therapy and paclitaxel, docetaxel, and irinotecan for second-line systemic therapy. Local therapy was delivered to all oligometastatic lesions of patients assigned to the systemic and local therapy group, followed by systemic therapy within 0–2 weeks of day 1 of local therapy. Local treatment modalities depended on the localization and size of the lesions, the nearby organs at risk, and previous treatments near the metastases, with the goal of achieving disease control while minimizing potential toxic effects^22^. The overall survival benefit was less profound in patients undergoing immunotherapy (HR 0.57; 95% c.i. 0.24 to 1.36), which is currently standard of care in patients with metastatic oesophageal cancer with programmed death-ligand 1 expression (that is, a combined positive score ≥ 1%)^24^. However, it should be noted that this study from China only included patients with oesophageal squamous cell carcinoma, whereas oesophageal adenocarcinoma is the predominant histological subtype of oesophageal cancer in Western populations^25^.

The prospective German phase II trial evaluated the value of surgery of the primary tumour and metastases in patients with genuine oligometastatic oesophageal junction or gastric adenocarcinoma^12^. In this prospective trial, patients first underwent four cycles of FLOT chemotherapy, and patients with a response at restaging underwent surgery of the primary tumour and metastases. Subsequently, patients underwent four cycles of postoperative FLOT chemotherapy. The overall survival of patients with a response to systemic therapy and surgery of the primary tumour and metastases was better than that of patients without a response to systemic therapy and no surgery of the primary tumour and metastases (31.3 *versus* 15.9 months)^12^.

*Table 1* provides an overview of ongoing and published trials in genuine oligometastatic oesophageal cancer.

**Table 1:** Overview of ongoing and published trials in oligometastatic oesophageal and gastric cancer

Author/sponsor name or ClinicalTrials.gov ID	Primary tumour	Country	Study phase and type	Maximum no. of organs/metastases	Type of genuine OMD	Staging	Treatment primary	Treatment metastases	Median overall survival
**Completed**									
Liu *et al*. (2023)^22^	Oesophageal SCC	China	II R	3/4	Synchronous and metachronous	CT	Surgery or SBRT	ChT ± IO + RT or surgery *versus* ChT ± IO	Median overall survival not reached after 30 months of follow-up *versus* 18.6 months
Liu *et al*. (2020)^13^	Oesophageal SCC	China	II NR	NS/3	Metachronous	CT or ^18^F-FDG PET	Surgery ± adjuvant RT ± ChT	SBRT ± ChT	24.6 months
Zhao *et al*. (2023)^23^	Oesophageal SCC	China	II NR	NS/5	Synchronous and metachronous	NS	NS	IO + ChT + SBRT	12.8 months
Al-Batran *et al*. (2017)^12^	Gastric AC or EGJ AC	Germany	II NR	1 + RPLN/organ-specific	Synchronous	CT/MRI or ^18^F-FDG PET	Surgery if resectable after ChT	ChT ± surgery + ChT	31.3 months
**Ongoing**									
NCT04510064 (Fudan University)	Gastric AC or EGJ AC	China	II NR	1/organ-specific	Synchronous	CT or MRI	Surgery if resectable after IO	IO + ChT + surgery	NA
NCT04248452 (ECOG-ACRIN Cancer Research Group)	Oesophageal AC and gastric AC	USA	III R	NS/3	Synchronous	CT or MRI	NS	ChT	NA
								ChT + SBRT	
NCT03161522 (MD Anderson Cancer Center)	Oesophageal AC	USA	II R	1/3	Synchronous	^18^F-FDG PET/CT	NS	ChT	NA
								ChT + SBRT or surgery	
NCT02578368 (FLOT5; Krankenhaus Nordwest)	Gastric AC or EGJ AC	Germany	III R	1 + RPLN/organ-specific	Synchronous	CT/MRI or ^18^F-FDG PET	Surgery in intervention group	ChT + surgery	NA
								ChT	
NCT04512417 (Zhejiang Cancer Hospital)	Oesophageal SCC or AC	China	II R	NS/4	Synchronous and metachronous	NS	NS	IO + SBRT	NA
								IO	
NCT06084897 (BEIR1; Cancer Institute and Hospital, CAMS)	Oesophageal SCC	China	II R	NS/NS	Synchronous and /metachronous	NS	Surgery or SBRT	ChT + consolidation RT	NA
								ChT + salvage RT	

OMD, oligometastatic disease; SCC, squamous cell carcinoma; R, randomized; CT, computed tomography; SBRT, stereotactic body radiation therapy; ChT, chemotherapy; IO, immuno-oncology; RT, radiotherapy; II NR, not randomized; NS, not specified; ^18^F-FDG, ^18^F-fluorodeoxyglucose; PET, positron emission tomography; AC, adenocarcinoma; EGJ, oesophagogastric junction; RPLN, retroperitoneal lymph node; MRI, magnetic resonance imaging; NA, not available; ECOG-ACRIN, Eastern Cooperative Oncology Group and American College of Radiology Imaging Network; CAMS, Chinese Academy of Medical Sciences.

#### Incidence and outcomes of induced OMD

Specific data regarding the incidence and outcomes after conversion surgery for induced OMD in oesophageal cancer are currently lacking. However, insights from a retrospective study, including primarily lung, prostate, melanoma, breast, or colorectal cancer patients, indicate that approximately 25% of patients with OMD have induced OMD^26^. The median overall survival for patients with induced OMD (24.1 months) was worse than that of patients with *de novo* OMD (46.3 months) or repeat OMD (50.3 months)^26^. These findings suggest that despite successful systemic therapy leading to the transition from polymetastatic disease to OMD, the induced OMD state may still exhibit unfavourable biological characteristics. However, it is important to note that although these results originate from a disease-agnostic study, their applicability to induced OMD in oesophageal cancer warrants consideration. Given the poorer overall survival associated with induced OMD compared with genuine OMD, it may be better to use stricter selection criteria for local treatment in induced OMD *versus* genuine OMD. One of these stricter selection criteria could be a longer duration of systemic therapy and a better response (for example, only offering local treatment to patients without progression after systemic therapy instead of also offering local treatment to patients with progression in size only, as is the case in genuine OMD, or using the clearance of ctDNA). Moreover, there may be selection bias in some studies of OMD treatment. Patients who are suitable for extensive treatment regimens, such as induction treatment and conversion surgery, tend to have fewer co-morbidities and a better performance status^12,22^.

## Gastric cancer

So far, no randomized clinical trials (RCTs) comparing conversion surgery to continued systemic therapy in gastric cancer patients with distant metastatic disease have been reported. Most studies are retrospective case series, although there are a few published non-randomized prospective studies, and several RCTs ongoing (see *Table 1*). Currently, conversion surgery aimed at radical (R0) resection for gastric cancer patients with limited distant metastatic burden, and with response to chemotherapy, is weakly recommended by international guidelines^27,28^. A longstanding problem for research in this field had been the lack of a widely accepted definition of OMD until the recent multidisciplinary European expert consensus statement from the OMEC group regarding the definition, staging modalities, and management of OMD in gastric and oesophageal cancer^16^. As mentioned above for oesophageal cancer, OMD with curative potential after conversion surgery was defined by the OMEC group as one organ affected with a maximum of three metastases, or one distant metastatic lymph node station involved and at least stable disease after systemic therapy^16^. Metastatic disease to the peritoneum was not included in the statement.

One of the few prospective studies published is the AIO-FLOT3 study from Germany^12^. AIO-FLOT3 is a non-randomized phase II study of gastric and gastro-oesophageal junction adenocarcinomas testing intense up-front triple chemotherapy using the FLOT regimen in three different cohorts: patients with operable non-metastatic disease; patients with limited (oligo) distant metastatic disease; and patients with widespread distant metastatic disease^12^. Among patients with limited distant metastatic disease, 60% could proceed to surgery with curative intent after completing chemotherapy, with a median overall survival of 31.3 months after surgery^12^. In a Chinese prospective cohort study^29^ comparing non-randomized patients with a limited single-site metastatic burden of gastric cancer undergoing surgery to those continuing with systemic therapy, after intense triple chemotherapy median overall survival was 18 months in the group continuing with systemic therapy but was not reached in the group that underwent conversion surgery after a median 30-month follow-up period. In that study^29^, given that non-randomized treatment allocation was likely to have been influenced by the response to systemic therapy, selection bias is likely to have affected the results. Both these studies^12,29^ included some patients with peritoneal metastases.

A third recently published non-randomized phase II trial^30^ included only gastric cancer patients with peritoneal disease, setting the limit at a peritoneal cancer index of ≤ 10. All patients had upfront systemic chemotherapy and a subset of patients also underwent neoadjuvant laparoscopic HIPEC. Patients with at least stable disease were then operated on, undergoing conversion gastrectomy with HIPEC and complete excision of all peritoneal disease. Median survival was 24.9 months from enrolment and 14.4 months from surgery^30^. The 3-year survival rate after surgery was 25%^30^.

The large retrospective multicentre CONVO-GC-1 study^31^ included more than 1200 patients from Japan, Korea, and China, and reported median overall survival of 36.7 months after conversion surgery and 56.6 months when R0 surgery was achieved. Other studies of stage IV gastric cancer^32–36^, in some cases also including initially unresectable locally advanced gastric cancer^37–39^, also demonstrated a survival benefit of conversion surgery compared with chemotherapy alone, with a median overall survival of 26.0–63.6 *versus* 14.0–18.8 months. However, the evidence value of these types of retrospective study is limited due to the high risk of selection bias and residual confounding of prognostically better patients being offered conversion surgery.

Subgroups of patients with liver metastases, distant lymph node metastases, ovarian metastases (Krukenberg tumours), peritoneal metastases, or positive peritoneal cytology have been evaluated in retrospective observational studies. In those studies, median overall survival ranged from 41.7 to 49.2 months for liver metastases^32,35^, from 33.5 to 54.3 months for distant lymph node metastases^31,35^, and from 13.6 to 19.0 months for ovarian metastases^32,40^. In patients with positive cytology but no macroscopic peritoneal metastases, high conversion rates of 49–64% following systemic chemotherapy have been reported^41–43^, with median overall survival of 30.4–42.4 months after conversion surgery^31,42,44^. Again, these cohorts are likely to have been highly selected.

Generally, metastatic gastric cancer can be characterized by the presence of peritoneal involvement, with distinct biological, diagnostic, and therapeutic properties of peritoneal metastatic disease. Conversion surgery for non-peritoneal gastric cancer metastases largely resembles that of other oligometastatic cancers; however, staging and therapy for peritoneal gastric cancer metastases are quite different, with the need for repeated staging laparoscopies and the increasing use of several types of still-investigational intraperitoneal chemotherapy, including HIPEC, intraperitoneal catheter-based chemotherapy (IPC), usually with taxanes, and pressurized intraperitoneal aerosol chemotherapy (PIPAC). Asian trials suggest that IPC using paclitaxel can soon be a part of the standard therapy arsenal^45,46^. IPC in these Asian studies^45,46^ showed little systemic toxicity, objective response rates approaching 90%, and promising results regarding overall survival and the proportion converted to radical resectability.

## Pancreatic cancer

The management of non-metastatic pancreatic ductal adenocarcinoma (PDAC) has historically been informed primarily by tumour anatomy. Pancreatic tumours have been defined as resectable (R), borderline resectable (BR), or locally advanced (LAPC) based on their anatomical relationship with the adjacent mesenteric vasculature. Various anatomical staging systems exist, which differ particularly regarding portal–mesenteric venous and coeliac axis involvement (*Table 2*). The National Comprehensive Cancer Network classification is probably the most widely implemented staging system, at least in the scientific community^52^. However, high interobserver variability may affect staging, significantly influenced by subjective interpretation of objective imaging criteria^53^. Although definitions differ between guidelines, disease stage increases with the extent to which a tumour contacts the major mesenteric vessels on radiographic studies^54^. Patients with R or BR PDAC are typically offered pancreatectomy with curative intent, when possible, either before or after systemic chemotherapy; historically, patients with LAPC have been considered unresectable and have rarely been offered surgery.

**Table 2:** Anatomical resectability criteria for pancreatic cancer according to different definitions

	NCCN (version 1.2022)^47^	AHPBA/SSAT/SSO (2009)^48^	MDACC (version 2021)^49^	Alliance trial (2013)^50^	JPS classification
					(7th edn 2016)^51^
**PRPC**	Arterial: CA/SMA/CHA: no arterial contact Venous: SMV/PV: no tumour contact or ≤ 180° contact without vein contour irregularity	Arterial: CA/HA/SMA: clear fat planes around Venous: SMV/PV: no radiological evidence of abutment, distortion, tumour thrombus, or venous encasement	Arterial*: SMA/CA: no interface between tumour Venous*: SMV/PV: patent confluence		Arterial: SMA/CA/CHA: no contact/invasion Venous: SMV/PV: < 180° contact or invasion without occlusion
**BRPC**	Arterial: Head/uncinate process: SMA: contact ≤ 180° CHA: contact without extension to CA or hepatic artery bifurcation, allowing for safe and complete resection and reconstruction Presence and degree of contact with variant arterial anatomy (RHA, CHA), because it may affect surgical planning Body/tail: CA: contact ≤ 180° CA: contact > 180° without involvement of Ao and intact and uninvolved GDA (permitting modified Appleby procedure) Any location: Venous: SMV/PV: contact > 180°, contact of ≤ 180° with contour irregularity of the vein or thrombosis of the vein but with suitable vessel proximal and distal to the site of involvement, allowing for safe and complete resection and vein reconstruction IVC: tumour contact	Arterial: SMA: abutment ≤ 180° of the circumference of the vessel wall GDA: encasement up to the HA with either short segment encasement or direct abutment of the HA, without extension to the CA Venous: SMV/PV: tumour abutment with or without impingement and narrowing of the lumen, encasement of the SMV/PV but without encasement of the nearby arteries, or short segment venous occlusion resulting from either tumour thrombus or encasement but with suitable vessel proximal and distal to the area of vessel involvement, allowing for safe resection and reconstruction	Arterial*: SMA: abutment ≤ 180° HA/GDA: short segment encasement abutment Venous*: SMV/PV: short segment occlusion and patent vessel above and below	Arterial: SMA/CA: contact < 180° of the circumference of the vessel wall CHA: reconstructible, short segment interface between tumour and vessel of any degree Venous: SMV/PV: contact ≥ 180° of the circumference of the vessel wall, and/or reconstructible occlusion	Arterial: SMA/CA: contact/invasion < 180° without stenosis or deformity CHA: tumour contact/invasion without contact or invasion of the PHA and/or CA Venous: SMV/PV: contact/invasion ≥ 180° or occlusion, not exceeding the inferior border of the duodenum SMA/CA/CHA: no contact/invasion
**LAPC**	Arterial: Head/uncinate process: SMA or CA: contact > 180° Body/tail: SMA or CA: contact > 180° CA and Ao: contact/involvement Any location: Venous: SMV/PV: unreconstructible due to tumour involvement or occlusion (due to tumour or bland thrombus)		Arterial: SMA or CA: interface between tumour > 180° Ao: interface with tumour Venous: Unresectable venous occlusion		Arterial: SMA/CA: contact/invasion ≥ 180° CHA: tumour contact/invasion with contact or invasion of the PHA and/or CA Ao: tumour contact or invasion Venous: Contact/invasion ≥ 180° or occlusion, exceeding the inferior border of the duodenum

^*^Low-risk features (no suspicion of metastatic disease, CA 19-9 ≤ 500 units/ml with normal bilirubin, manageable and optimized co-morbidities) or high-risk features (suspicion of metastatic disease, CA 19-9 > 500 units/ml with normal bilirubin, reversible and optimizable co-morbidities). NCCN, National Comprehensive Cancer Network; AHPBA, The Americas Hepato-Pancreato-Biliary Association; Ao, aorta; SSAT, society for surgery of the alimentary tract; SSO, society of surgical oncology; MDACC, MD Anderson Cancer Center; JPS, Japan Pancreas Society; PRPC, primary resectable pancreatic cancer; CA, celiac artery; SMA, superior mesenteric artery; CHA, common hepatic artery; SMV, superior mesenteric artery; PV, portal vein; BRPC, borderline resectable pancreatic cancer; GDA, gastroduodenal artery; HA, hepatic artery; RHA, right hepatic artery; IVC, inferior vena cava; LAPC, locally advanced pancreatic cancer; PHA, proper hepatic artery; CA 19-9, carbohydrate antigen 19-9.

The term conversion surgery has been increasingly used to describe potentially curative operations performed after neoadjuvant therapy for PDAC, particularly for LAPC^55^. In this regard, four points must be noted. First, the conversion of anatomy from technically unresectable to resectable that may occur using this strategy is due to preoperative therapy rather than pancreatectomy (that is, ‘conversion chemotherapy’ may be a preferable term in this context). Second, the decision to operate on patients with PDAC is subjective, can be subject to internal and external biases, and may change over time. Thus, the perceived effects of neoadjuvant therapy on tumour anatomy are commonly overstated, particularly when evaluated retrospectively^50^. Third, patients with localized PDAC are increasingly staged not only on the basis of the anatomy of their primary tumours but also on their own physiological status and the anticipated natural history of their cancers, because it is known that the biology of pancreatic cancer has a greater effect on patient outcomes than anatomical presentation, and each patient's co-morbidities and performance status are associated with their ability to undergo resection and to tolerate ongoing therapy. This concept was introduced in 2008 by Katz *et al*.^54^, with a three-category classification for BR PDAC including borderline resectability by vascular involvement (MD Anderson Type A), by suspected extrapancreatic disease (MD Anderson Type B), and by marginal performance status or severe co-morbidities (MD Anderson Type C). The A-B-C nomenclature for BR PDAC was later incorporated by an international expert consensus in 2017^56^, which defined biologically BR PDAC as the presence of preoperative carbohydrate antigen (CA) 19-9 levels > 500 U/ml and/or regional lymph node metastasis, and conditional BR PDAC as an ECOG performance status grade ≥ 2. In 2019, the American Society of Clinical Oncology guidelines^57^ emphasized that anatomical staging should not be the leading criterion in decision-making because biological and conditional factors are increasingly acknowledged to be primary parameters for patient selection. The shift from an anatomically towards a prognosis-based resectability (also including biological and conditional criteria) has been recently highlighted in the new REDISCOVER international guidelines on the perioperative care of BR pancreatic cancer and LAPC^58,59^.

Finally, neoadjuvant therapy is now often administered to patients across all three anatomical stages of localized PDAC. Thus, the use of surgery after neoadjuvant therapy is highly dependent on the clinical context in which it is performed. If the term ‘conversion surgery’ is to be used to describe operations conducted after neoadjuvant therapy for PDAC, this context must be clearly communicated and documented.

### Resectable and borderline resectable PDAC

The use of the term conversion surgery to describe operations performed after neoadjuvant therapy for patients with R or BR PDAC is inappropriate. Although patients with such tumours may be offered surgery *de novo* followed by treatment with 6 months of modified FOLFIRINOX (FFX; fluorouracil, leucovorin, irinotecan, and oxaliplatin), gemcitabine with capecitabine, or gemcitabine or fluorouracil alone, treatment with neoadjuvant therapy consisting either of modified FFX or gemcitabine and nab-paclitaxel (GNP) in anticipation of subsequent pancreatectomy is now common^60–64^. In patients whose tumours may be reasonably resected even without previous therapy, the goal of neoadjuvant therapy is not the conversion of tumour anatomy from unresectable to resectable; rather, the aims of neoadjuvant therapy are to treat the occult systemic disease presumed to exist in all patients, maximize the dose intensity of chemotherapy when it may best be tolerated, and select patients responsive to therapy for whom pancreatectomy may be most appropriate. Exercise, nutrition, and psychiatric counselling may all be prescribed concurrent with neoadjuvant therapy to convert patients from inoperable to operable through physiological prehabilitation.

Although the primary intent of neoadjuvant therapy in these settings is not conversion, neoadjuvant therapy does appear to have measurable effects, including increasing R0 resection rates, reducing lymph node metastasis, and perhaps even prolonging survival^63,65–68^. Chemotherapy is an essential component of neoadjuvant therapy in these settings, with clinical equipoise between modified FFX and GNP^64,65,69^. However, the role of RT is less clear. After a long-term follow-up of patients enrolled in the Dutch PREOPANC-1 trial^70^, the 5-year overall survival rate of patients with R and BR PDAC was significantly greater among those who received neoadjuvant gemcitabine-based chemoradiotherapy than among those who underwent pancreatectomy de novo (20.5 vs. 6.5%, respectively). In the Alliance A021501 trial^71^, preoperative FFX, but not preoperative FFX followed by hypofractionated RT, was established as a reference regimen for anatomically BR pancreatic cancer. In addition, the preliminary results of PREOPANC-2^72^ suggest equipoise between preoperative FFX and gemcitabine-based chemoradiotherapy. The recently published NORPACT-1 trial^73^ was unable to show a survival benefit with neoadjuvant FFX compared with upfront resection in R PDAC, although there were challenges associated with the administration of FFX in the neoadjuvant setting. The PREOPANC-3^74^ and Alliance A021806^75^ trials are also assessing perioperative FFX *versus* upfront resection with adjuvant FFX.

### Locally advanced PDAC

Historically, resection of LAPC was uncommon due to the morbidity associated with the arterial reconstructions often required and the poor overall survival following even microscopically complete resections^76,77^. Recently, however, there has been renewed interest in surgery in this setting. Retrospective studies of patients who underwent pancreatectomy for LAPC after neoadjuvant therapy describe acceptable morbidity, mortality, and survival rates^76–79^. Despite the proliferation of these retrospective analyses, the role of surgery for LAPC is more limited than it is for R or BR PDAC, even after neoadjuvant therapy. In prospective trials^80–83^, 4–36% of LAPC patients treated with a variety of regimens have undergone subsequent resection, with R0 rates of these resected patients ranging from 25 to 67%. However, it is important to recognize that the resection rates in these studies were likely influenced as much by study design as by the treatment regimens evaluated. For example, both LAPACT^81^ and NEOLAP^82^ (resection rates 16 and 32%, respectively) enrolled patients who had locally advanced disease as determined by intraoperative assessment as well as on the basis of standardized radiographic criteria. NEOLAP also included patients staged as BR^82^. Among these four prospective trials^80–83^, only CONKO-007^80^, with a resection rate of 36%, enrolled patients with LAPC robustly staged using radiographic criteria and judged by an independent surgical board. Nonetheless, important lessons have emerged from these studies. First, at least some patients with LAPC do appear to benefit from pancreatectomy following chemotherapy and/or chemoradiation. Second, as always, margin-negative resection should be the goal of surgery. In the NEOLAP trial^82,84^, patients who underwent R0 resection had a median overall survival of 40.2 months compared with 17.1 months among those who underwent R1 resection, with the latter being similar to that of patients who did not undergo surgery at all. Methods to prospectively discriminate between patients who will benefit from surgery and those who will not must be a critical focus of investigation. Finally, although RT does not appear to prolong survival in this setting relative to chemotherapy alone, it does seem to improve locoregional control. Even among patients with LAPC, the extent to which neoadjuvant therapy is converting these patients from unresectable to resectable, rather than selecting for favourable biological features is unclear; in reality, chemotherapy and/or chemoradiation therapy may be serving both functions.

### Treatment response

Relatively few studies have explored changes in anatomical relationships in response to preoperative therapy. Initially, significant anatomical downstaging was uncommon after treatment with gemcitabine-based chemotherapy^68^. However, more recently it was demonstrated that significant anatomical changes may occur after multiagent chemotherapy with or without subsequent chemoradiation. Among 141 patients with localized PDAC evaluated retrospectively, 31% had a partial response according to Response Evaluation Criteria in Solid Tumours (RECIST)^85^ after neoadjuvant therapy and 29% of patients with BR pancreatic cancer or LAPC were downstaged^86^. Radiographic downstaging was more common after induction FFX or GNP than after subsequent chemoradiation (24 *versus* 6%; *P* = 0.04).

Another study^87^ classified LAPC into three different categories with a spectrum of venous and arterial involvement and showed that improvement between these categories during treatment was significantly correlated with improved survival. On contrast-enhanced CT, a reduction in tumour size, either measured volumetrically or by RECIST criteria, is associated with the response measured histopathologically^88^. A reduction in the maximum standard uptake value on ^18^F-FDG PET may also suggest a pathological response^89^. Using a CT-based radiomic signature, a prospective trial^90^ of patients with high-risk BR PDAC treated with neoadjuvant therapy (modified FFX) and chemoradiation found that the CT-based biomarker was associated with both resection and survival. Together, these studies suggest that FOLFIRINOX or GNP, whether followed by chemoradiation or not, may facilitate resection in up to one-third of patients with LAPC.

Histopathological analysis of the surgical specimen is the standard used to measure the response to therapy. However, this cannot be performed in real time, so cannot be used to inform either the type or duration of neoadjuvant therapy^88^. At present, CA 19-9 is the most clinically relevant biomarker. CA 19-9 dynamics should be followed during the neoadjuvant period; a reduction in CA 19-9, preferably to normal levels, is a favourable prognostic indicator^78,88^. Dynamics associated with short survival include high pretreatment CA 19-9 levels, CA 19-9 levels that do not fall, or CA 19-9 levels that rise over the course of neoadjuvant therapy^66,91^. Among patients who do not express CA 19-9, Duke pancreatic monoclonal antigen type 2 (DUPAN-2) has emerged as an additional biomarker; carcinoembryonic antigen and cancer antigen 125 can also be measured^92,93^. Novel biomarkers, such as ctDNA, are also being evaluated as a method of monitoring the biological response to therapy^94^.

Finally, changes in patients’ symptoms should also be considered as treatment response criteria. Improvements in PDAC-related symptoms can indicate a response to therapy. Patient factors, such as performance status, weight loss, and sarcopenia, should be evaluated frequently during neoadjuvant therapy^95–98^. Deterioration should prompt attempts at remediation before pancreatectomy is considered.

### Technical elements

Before pancreatectomy, virtually all patients should be evaluated laparoscopically during their neoadjuvant therapy to search for radiographically occult metastatic disease to the liver or peritoneal surfaces^52,99^. In an analysis of over 1000 patients undergoing staging laparoscopy^100^, 18% were found to have gross peritoneal metastases and/or peritoneal cytology. Magnetic resonance imaging (MRI), particularly with diffusion-weighted imaging, has also shown advantages in detecting small/occult liver metastases compared with standard radiology, as confirmed by a recent systematic review and meta-analysis^101^, which found that MRI with diffusion-weighted imaging significantly improved the detection of occult liver metastases compared with contrast-enhanced CT, potentially preventing non-therapeutic surgeries.

Surgeons performing pancreatectomy for LAPC after neoadjuvant therapy should have a plan for venous and arterial reconstructions. Single-centre series^102,103^ have reported encouraging results, with superior mesenteric artery resection and reconstruction and mortality consistently below 5% at 90 days, albeit after a learning curve of around 100 cases. However, tumours with hepatic or superior mesenteric artery involvement can occasionally be divested from the arteries rather than requiring arterial resection. Arterial divestment may reduce morbidity and mortality compared with arterial resection, while concomitantly increasing the risk of R1 resection rates^104^. The NEOLAP trial^82^ demonstrated that R1 resections for LAPC have a similar prognosis to no surgery, raising questions about the utility of less-risky but potentially non-therapeutic R1 resections. However, it is important to consider that achieving an R1 resection can still provide benefits over non-surgical management. Studies^105,106^ have shown that patients with R1 resections have better survival outcomes than those who do not undergo surgery at all, particularly when neoadjuvant therapy is used. Therefore, as a less morbid alternative to arterial resection, divestment can be considered an alternative in select patients to optimize outcomes.

For patients who ultimately do not proceed to surgery, other local therapy options are available. These therapies include intraoperative RT, brachytherapy, SBRT, and other local ablative techniques^107^. Few prospective trials have evaluated differences in the efficacy of these treatments. Comparing local ablative therapies to resection in patients with advanced disease, one retrospective study^108^ did find similar local control, with worse overall survival with ablative therapies.

### Oligometastatic PDAC

A commonly accepted definition for oligometastatic PDAC is currently lacking. A recent systematic review^109^ identified 76 studies reporting definitions and/or local consolidative treatment for oligometastatic PDAC. Definitions varied widely, but the definition of oligometastasis was mostly characterized by metastasis in a single organ, whether three or fewer synchronous lesions in the liver or two or fewer metachronous lesions in the lungs (*Fig. 2*)^109^. Regardless of the preferred definition, the role of pancreatectomy in the setting of OMD is evolving. The largest series available was recently published by the Heidelberg group^110^, and included 173 patients who underwent surgical exploration after chemotherapy for metastatic PDAC between 2006 and 2019. In that study^110^, 93 patients underwent resection of the primary tumour and metastatic sites; of these patients, 45 achieved complete pathological response of metastases (yp (post-therapy) M0), whereas 48 had residual metastases (ypM1). Overall survival was significantly higher in the ypM0 group (25.5 months) than in the ypM1 group (10.7 months) and in those without resection (8.1 months)^110^. In addition, adjuvant chemotherapy was associated with prolonged survival in resected patients, particularly in the ypM0 subgroup^110^. Multivariable analysis identified conversion surgery, CA 19-9 levels, and time of resection as independent prognostic markers^110^.

However, pancreatectomy should be used very selectively in the setting of synchronous or metachronous metastatic disease at present. Resection of metastatic disease should not be informed solely by the number of metastases, but should also consider biological features, such as response to therapy. A prolonged period of progression-free survival on chemotherapy should be observed before considering resection. According to Hank *et al*.^110^, timing of resection between 5 and 9 months after diagnosis was an independent predictor of longer overall survival. The feasibility and safety of metastasectomy should be carefully considered. Location should also be considered, because lung metastases often have a more favourable prognosis than peritoneal or liver metastases^111,112^. The role of local ablative therapies to metastases was investigated in the EXTEND trial^113^, with the results soon to be reported. In that phase II multicentre open-label parallel group randomized basket trial^113^, the addition of metastasis-directed therapy (MDT) in addition to SOC *versus* systemic therapy alone was evaluated. Patients with five or fewer sites of metastatic disease and pancreatic cancer were included. The local therapies used included RT and radiofrequency ablation (RFA). The primary endpoint was progression free survival (PFS). An HR of 0.43, which was statistically significant (*P* = 0.03), favoured MDT plus SOC over SOC alone. After a median follow-up of 17 months, the probability of PFS in the MDT arm was 42%, compared with 8.6% in the SOC-only arm (median PFS 10.3 *versus* 2.5 months)^113^. These results suggest that well-controlled OMD with MDT could be amenable to subsequent surgical control in PDAC.

## Hepatocellular carcinoma

**Fig. 4:**
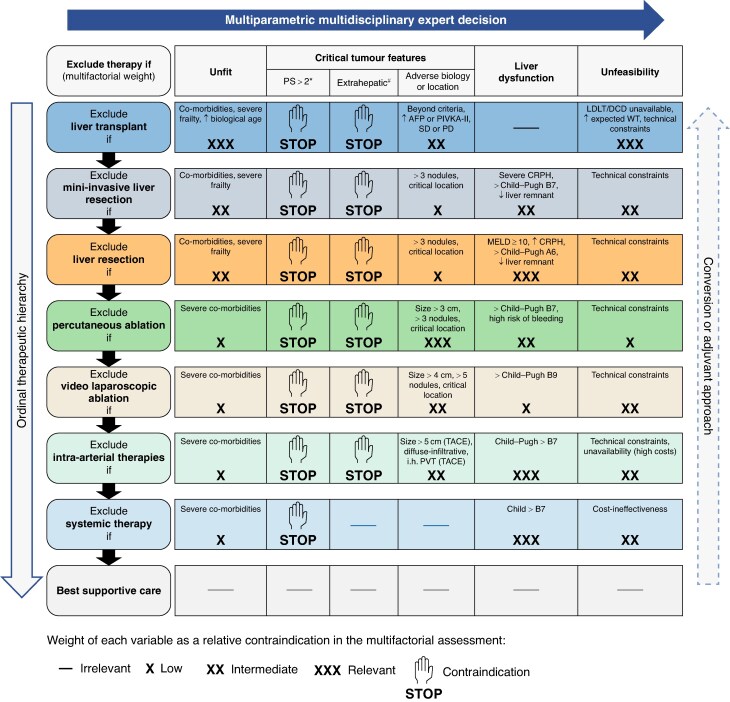
Multiparametric and converse therapeutic hierarchy for hepatocellular carcinoma This figure is derived from Altmayer *et al.*^101^ and Napoli *et al.*^103^. *PS is an indicator of tumour-related symptoms, and therefore tumour aggressiveness. #Includes extrahepatic metastases and invasion of the main trunk of the portal vein or inferior vena cava. The arrow on the right indicates the converse therapeutic hierarchy (conversion or adjuvant approach); note that the evidence supporting this concept is still weak. PS, performance status; AFP, α-fetoprotein; PIVKA-II, protein induced by vitamin K absence II; LDLT, living donor liver transplantation; DCD, donor after circulatory death; DBD, donor after brain death; MELD, model for end-stage liver disease; CRPH, clinically relevant portal hypertension; TACE, transarterial chemoembolization; PVT, portal vein thrombosis.

Treatment allocation is particularly complex in patients with HCC due to the prognostic impact of the underlying liver disease and the high prevalence of clinical frailty in these patients^114^. Fortunately, there are multiple treatment options for HCC. Some of these options are considered potentially curative: liver transplantation (LT), liver resection (LR), and percutaneous or surgical ablation. Other options, such as intra-arterial therapies or systemic therapies, have proved to improve the survival of patients with HCC^114^. Therefore, when the aim is to cure a patient with HCC, an expert multidisciplinary team should evaluate these therapeutic options in a hierarchical order^115^. The therapeutic hierarchy concept avoids the risk of undertreatment inherent to a rigid-stage hierarchical approach, such as that proposed by the Barcelona Clinic Liver Cancer algorithm^114^. Conversely, to overcome the potential risk of overtreatment intrinsic to therapeutic hierarchy, an accurate multiparameter evaluation of each treatment option should be undertaken by an expert tumour board^114,116^ (*Fig. 4*). When a curative aim is excluded (that is, surgical options or ablation are contraindicated), the expert multidisciplinary team should change their focus to the possibility of regaining the chance for curative therapy. This new clinical perspective is termed converse therapeutic hierarchy (*Fig. 4*, right-hand arrow), indicating the possibility of converting a tumour that is unsuitable for curative treatment to one with a new chance for a cure. The term ‘converse therapeutic’ means that the direction is from non-curative to remedial therapeutic options. However, this term also indicates that the hierarchical order of HCC treatments is inverted when the aim is conversion. From this novel perspective, the highest rank position is occupied by systemic therapies, followed by, or in combination with, intra-arterial therapies. The conversion involves a change in tumour biology through therapies that intervene at a systemic level, potentially on all tumour cells (that is, both in known tumour sites and, likely, in subclinical metastatic sites)^117^. For this reason, systemic therapies are at the top of the converse therapeutic hierarchy when truly biologically effective.

This concept is particularly true today when new effective systemic therapies are available. The objective response rate in phase 3 clinical trials has risen from 10–11% with sorafenib to 25–30% with lenvatinib or immunotherapy^118–121^.

Regarding transplantation, the concept of conversion coincides with the downstaging concept and is well consolidated by evidence from the literature^122–133^. Most of the evidence currently available regarding the results of this practice comes from uncontrolled studies showing that downstaging, if effective in reducing tumour mass, is associated with post-transplant survival rates similar to those obtained in patients who comply *ab initio* to transplant selection criteria adopted by a given centre. A multicentre RCT was recently published comparing the survival of patients undergoing transplantation after effective downstaging to that of a control group undergoing non-transplant therapies^134^.

It is possible to distinguish two categories of downstaging: relative and absolute. Relative downstaging aims to bring the patient to within the transplant criteria adopted by the centre. In most published studies^122–126^, patients were considered eligible for transplant when downstaging reported the disease stage within the Milan criteria, starting from the intermediate/advanced stages and in the absence of vascular or biliary invasion. Therefore, this type of relative downstaging can also be defined as morphological. However, recent US studies^126,127^ have highlighted the prognostic role of α-fetoprotein (AFP) in downstaging. A reduction in AFP levels below predefined values (500 or 100 ng/ml) is associated with transplant results similar to those obtained in patients within the Milan criteria *ab initio*, regardless of AFP levels before the start of locoregional therapies^126^. This underlines the importance of considering indicators of biological aggressiveness when evaluating the response to treatment and selecting patients.

Conversely, absolute (or biological) downstaging aims to obtain an effective (partial or complete) and persistent response to locoregional or systemic therapies, regardless of pre-established arrival criteria. This strategy was came out of the retrospective study by Otto *et al*.^135^, in which patients with a good partial response to chemoembolization (transarterial chemoembolization) had a 5-year survival rate after transplantation that was comparable to that of the control group (patients within the Milan criteria *ab initio*), regardless of whether they met the Milan criteria or not. Subsequent studies^134–137^ have confirmed that the response to locoregional therapies can represent one of the critical factors in the transplant selection process of patients with HCC because of its ability to identify those with more favourable tumour biology, which leads to low rates of post-transplant relapse.

In addition, the comparators for the two types of downstaging are different. Although the comparator for relative downstaging is LT within the established criteria, the comparator for absolute downstaging is the alternative non-LT treatment^138^. An Italian consensus on allocation in LT^139^, a recent ‘position paper’ of the Italian Association for the Study of the Liver^140^, and the latest Italian HCC guidelines^141^ have established that downstaging for LT should be absolute/biological. From this biological perspective, to achieve an effective conversion to LT, it is essential to obtain a complete or partial response according to modified RECIST criteria^142^ and, at the same time, an effective response in terms of AFP levels. That is, an effective conversion to LT needs a response in terms of a reduction in viable HCC (that is, showing uptake in the arterial phase of contrast-enhanced radiological imaging) and not necessarily tumour shrinkage. Nonetheless, the crucial issue remains overall LT benefit, defined as net survival, which is calculated by subtracting survival obtainable with non-transplantation options from expected survival achieved with LT^143^.

The pathophysiological situation seems quite different when the aim is to convert unresectable HCC to LR. According to a recent consensus by Chinese experts^144^, unresectable HCC can be defined as that due to either surgical or oncological causes. Surgical causes refer to an inability to perform a safe surgical resection, and oncological causes refer to the predicted efficacy after surgery, which does not surpass other non-surgical treatment methods. The goal of conversion therapy is to eliminate these two causes and achieve a conversion from unresectable to resectable HCC. Tumour biology and aggressiveness should be accurately stratified to avoid early severe recurrence after potentially radical therapy^145^. Conversely, the capability of completing the process of conversion to surgery is intrinsically a surrogate of biological aggressiveness. Moreover, in addition to a biological conversion of the tumour, the concept of conversion to LR includes a technical conversion, which means tumour shrinkage. For this reason, the primary evaluation standard during the conversion period to LR should be the objective response rate based on RECIST 1.1^85^ criteria (not modified RECIST criteria^142^) and aggressive, multimodal treatments should be used to preserve liver function while maximizing the objective response rate. Some evidence, mainly from Asian countries^144^, suggests that hepatic artery infusion chemotherapy (HAIC) and selective internal radiation therapy may also have a role in tumour shrinkage for HCC conversion to curative LR.

From a more general perspective, treatments for liver parenchyma aimed at increasing technical resectability also fall within the concept of conversion therapies, such as procedures to improve remnant liver volume (that is, portal vein embolization, double vein embolization, associating liver partition and portal vein ligation for staged hepatectomy) or antiviral therapies to decrease the risk of post-resection liver dysfunction. The high complexity of HCC conversion to LR and the lack of solid evidence explain why this concept is yet to be acknowledged in Western hepatological guidelines^146^, even though the idea of downstaging has been brought to LT. A meta-analysis of observational studies^147^ suggests that a triple-combination approach with locoregional therapy, lenvatinib, and immunotherapy in patients with unresectable intermediate HCC yields a conversion rate to LR of approximately 40%. Therefore, a certain percentage of patients who were initially considered not amenable to curative resection may subsequently qualify for this approach. However, the broad application of these combinations to promote the conversion of non-surgical candidates to resectable hinges on the results of ongoing RCTs^148^.

## Colorectal liver metastases

Determining the optimal treatment approach for patients with initially locally untreatable colorectal liver metastases (CRLM) is challenging due to the absence of uniform criteria for (un)resectability/ablatability. Even among expert liver surgeons, there is a high level of disagreement^149,150^. In the majority of patients (approximately 80%), CRLM is locally untreatable at the time of diagnosis^151,152^. Considering that local treatment of CRLM is the main determinant of long-term survival and even cure, there is a major emphasis on increasing the number of patients eligible for treatment through various conversion strategies, including optimal induction systemic therapy and the implementation of structured re-evaluation of resectability and ablatability^153,154^. However, not all patients who may benefit from curative-intended local treatment are offered this option, which highlights the importance of the proper assessment of treatability in the setting of a multidisciplinary hepatobiliary team^155–161^.

### Conversion chemotherapy

In earlier studies^162–165^, downstaging from initially unresectable CRLM to a treatable state resulted in 5-year survival rates similar to those of patients with initially resectable CRLM. Conversion to resectability following systemic chemotherapy has been reported to range between 7 and 60%^151,166,167^, but the results are difficult to interpret due to inconsistent definitions of unresectability, various therapeutic regimens, previous treatment, and no consideration of combination with thermal ablation to achieve treatability. Current oncological conversion strategies are extensively accounted for in international guidelines^168,169^, which advocate doublet or triplet cytotoxic therapy with the addition of a targeted agent as the best treatment choice. In patients with left-sided RAS wild-type primary tumours, anti-epidermal growth factor receptor monoclonal antibodies are more effective than bevacizumab-based combinations. The combination of triplet cytotoxic therapy plus bevacizumab is considered most effective in patients with RAS-mutant disease. Local liver-directed treatment should be performed as soon as the CRLM is treatable to avoid unnecessary chemotherapy-induced liver injury. The best therapeutic regimens in initially unresectable CRLM were recently evaluated in the CAIRO5 trial from the Dutch Colorectal Cancer Group^170^, with significantly longer progression-free survival and higher complete local treatment following FOLFOXIRI (5-fluorouracil/leucovorin, oxaliplatin, and irinotecan)–bevacizumab in patients with a right-sided tumour site or RAS- or BRAF^V600E^-mutated primary tumours compared with doublet cytotoxic therapy plus bevacizumab. In addition, there was no difference in median progression-free survival with the addition of panitumumab to FOLFOXIRI instead of bevacizumab, but panitumumab was associated with more toxicity^170^. Even though there are no published RCTs comparing conversion rates from modern systemic chemotherapy alone to HAIC plus modern systemic chemotherapy, prospective studies^171–173^ report a conversion rate of between 33.3 and 52%. Whether HAIC improves conversion and survival in patients with unresectable CRLM is currently being evaluated in the Norwegian EXCALIBUR-1 + 2 trial (NCT04898504, NCT04840186) and in a US RCT comparing HAIC with SOC chemotherapy to chemotherapy alone in patients with unresectable CRLM after 3–6 months of first-line chemotherapy.

### Surgical and interventional radiological conversion strategies

The concept of resectability in CRLM has undergone a paradigm shift in recent decades, fundamentally affecting all liver surgery. Previously, resectability was defined by tumour factors such as tumour size, number of tumours, and bilobar distribution^174,175^. However, the current resectability limits are primarily defined by the amount of liver left after the resection^176^. Because post-hepatectomy liver failure (PHLF) is still the leading cause of mortality after modern liver surgery^177,178^, enough functional liver needs to be left after resection to avoid this condition. Several studies have demonstrated that the risk of PHLF increases as the size of the remnant liver decreases^179,180^. Hence, preoperative measurement of future liver remnant (FLR) volume has become instrumental for preoperative planning when a major LR is needed^181^. This is primarily performed through volumetric measurement of the FLR on preoperative contrast-enhanced CT and related to the total liver volume (either measured or estimated using various formulas)^182,183^. In patients with CRLM and healthy background liver, many studies^184,185^ report that resections are safe if the remnant liver is between 20 and 30% of the total liver volume. In patients with chemotherapy-induced liver injury, it is considered that an FLR over 30% is needed to avoid PHLF^184^.

Different techniques have been introduced to increase the number of patients who can undergo resection by inducing a regenerative response in the FLR^178^. Initially, two-stage hepatectomy was presented as a method to resect bilobar CRLM with small FLR^186,187^. This was then complemented with selective portal vein occlusion to induce additional growth of the FLR. This could be done by surgical ligation of the portal vein (typically the right main branch) in the first of the two stages^188^. Another method is to perform this selective occlusion by portal vein embolization (PVE), either as part of a two-stage procedure or before a one-stage operation^188^. In 2012, a new procedure, named associating liver partition and portal vein ligation for staged hepatectomy (ALPPS), was introduced^189^. This two-stage procedure combined liver transection and selective portal vein ligation in the first stage to achieve faster and more pronounced hypertrophy of the FLR. Consequently, resection rates were higher with this procedure than with PVE, but morbidity and mortality were also significantly higher^190,191^. In the context of CRLM, ALPPS appears to be associated with a lower risk, as demonstrated in several studies^192,193^. Due to the increased risk of PHLF and mortality associated with ALPPS, another method has emerged as a promising alternative. When PVE is combined with simultaneous hepatic vein embolization, increased and rapid hypertrophy has been observed^194^. This concept is currently being investigated in several prospective trials, including only patients with CRLM (NCT05428735)^195^.

Parenchymal-sparing liver surgery is another concept used in CRLM to enable resection in more patients^196^. This method is advocated by many liver surgeons because up to 70% of patients who undergo resection for CRLM will experience recurrence^197^, and performing major resections many times precludes a subsequent resection if too much liver has already been removed in a previous resection. Some staunch proponents of this strategy have further pushed the technical limits of this approach, enabling very advanced parenchymal resections^198^. In highly selected patients with unresectable liver-only colorectal metastases, LT provides a survival benefit over palliative chemotherapy, with 5-year overall survival rates as high as 60–80% using stringent selection criteria^199,200^. The TRANSMET trial^201^, a multicentre open-label prospective RCT, recently evaluated the impact of LT plus chemotherapy *versus* chemotherapy alone in patients with permanently unresectable CRLM. The study included 94 patients who were randomized to either LT plus chemotherapy or chemotherapy alone, and the primary endpoint was 5-year overall survival. The TRANSMET trial demonstrated a striking improvement in 5-year overall survival for the LT plus chemotherapy group, with a survival rate of 56.6%, compared with 12.6% in the chemotherapy-alone group (HR 0.37; *P* = 0.0003), in the intention-to-treat population^201^. In the per-protocol analysis, 5-year overall survival was even higher in the LT plus chemotherapy group, at 73.3%, compared with 9.3% in the chemotherapy-alone group^201^. Serious adverse events were observed in 80% of patients who underwent LT and in 83% of those treated with chemotherapy alone^201^. These findings suggest that LT plus chemotherapy significantly improves survival in select patients with permanently unresectable CRLM, supporting the validation of LT as a new standard option for these patients.

### Advanced ablation methods

Thermal ablation is another tool in the quest for more minimally invasive parenchyma-sparing treatments, where the latter have shown a favourable perioperative outcomes compared with open LR with similar long-term outcomes^202–204^. Through the destruction of metastasis locally, a maximum of surrounding tissue is preserved, reducing treatment-related morbidity and facilitating the retreatment of frequently recurring CRLM^205^. The most frequently applied local ablation treatment is thermal ablation, including microwave ablation and RFA, where cancer cells are locally destroyed using thermal energy. The success of thermal ablation ultimately depends on ablation energy expansion and the resulting ablation volumes adequately covering the targeted metastasis. For unresectable CRLM, the highest level of evidence for thermal ablation originates from the EORTC-CLOCC trial in which patients were randomly assigned to systemic treatment alone or systemic treatment plus RFA (with or without additional resection), showing prolonged survival^206^ and improved progression-free and overall survival in the long-term follow-up study^207^. In the case of extensive distribution of bilobar CRLM, combining thermal ablation with resection can preserve FLR and achieve 5-year overall survival rates of 37–56%, comparable to two-stage hepatectomy but with improved perioperative outcomes^208–211^. A sequential treatment strategy, with planned incomplete resection followed by postoperative image-guided completion of the ablation of intentionally untreated metastases, has been proposed^212^, supposedly providing improved local tumour control compared with intraoperative ultrasound-guided thermal ablation. Even in patients with unresectable CRLM, the combination of surgical resection and thermal ablation has been shown to provide overall survival rates that are comparable to those of patients with resectable CRLM treated with hepatectomy alone^213–215^.

High-intensity focused ultrasound is a technique where high power and high frequency ultrasound are bundled to deliver a thermal effect and locally destroy targeted metastatic tissue through coagulative necrosis. For unresectable CRLM, two recent trials^216,217^ reported local control rates between 50 and 80%.

Irreversible electroporation (IRE) is a non-thermal local ablation technique that spares the extracellular matrix of structures rich in stromal tissue, such as blood vessels and bile ducts, thereby enabling treatment of CRLM in the vicinity of vital hilar structures. For CRLM not treatable with resection or thermal ablation, primarily due to size and an intrahepatic location of recurrent CRLM, IRE has proven to be effective and relatively safe for CRLM ≤ 5.0 cm, with a median survival of 2.7 years from the first IRE^218^. As of now, IRE should be considered for CRLM that is truly unsuitable for thermal ablation and/or resection^219^.

Three ongoing RCTs aim to establish the role of stereotactic body radiation therapy (SBRT) in unresectable CRLM, comparing SBRT to microwave ablation (NCT04081168, NCT03654131, and NCT02820194). Although transarterial chemoembolization is a well-established treatment modality for primary liver cancer, its use in the treatment of CRLM is controversial, with very few reports in the conversion setting.

## Biliary tract cancer

Biliary tract cancer (BTC) represents a heterogeneous group of aggressive malignancies arising from the biliary epithelium. BTC includes distinct anatomical subtypes: cholangiocarcinoma (CCA), which is classified as intrahepatic (iCCA), perihilar (pCCA), and distal; and gallbladder cancer. Each of these has unique genetic aberrations and clinical manifestations. The prevalence of unresectable BTC at diagnosis highlights the need for effective therapeutic strategies^220^. Despite advances in surgical techniques and systemic therapies, the prognosis remains dire, with a 5-year survival rate below 10% for all stages combined^221^. Radical resection with negative margins represents the only curative option, although it is applicable to a minority of patients^220^. Conversion surgery, involving neoadjuvant therapies aimed at downstaging tumours to facilitate surgical resection, represents a significant advance in the treatment paradigm of this challenging disease.

### Current therapeutic strategies

Current therapeutic strategies for CCA emphasize a comprehensive multidisciplinary approach, integrating surgical intervention, oncological therapies, and advances in targeted therapy and immunotherapy^222^. In the realm of systemic therapy, regimens building on the backbone of gemcitabine and cisplatin have emerged as the standard first-line treatment, extending survival rates in the advanced stages of CCA^223^. In recent years there have been significant strides in the field of molecularly targeted therapies, with drugs designed to inhibit specific pathways implicated in tumour growth and progression. Agents targeting genetic aberrations, such as like fibroblast growth factor receptor 2 (FGFR2) fusion or rearrangement, mutations in isocitrate dehydrogenase (NADP^+^) 1 (*IDH1*) and isocitrate dehydrogenase (NADP^+^) 2 (*IDH2*), or HER2 amplification, have shown promise in clinical trials^222^, tailored to the tumour's molecular profile. Furthermore, the integration of RT with chemotherapy has shown promise as a potent therapeutic strategy^224^. This combination synergistically enhances the cancer-killing effects, offering a valuable option for patients with advanced or marginally resectable disease. Chemoradiation can also serve as a neoadjuvant treatment to downstage tumours, potentially converting previously inoperable tumours to resectable tumours, thereby opening avenues for curative surgical resection. The advent of novel surgical techniques and improvements in preoperative imaging have expanded the resectability criteria, offering hope to a broader patient cohort.

### Role of conversion surgery

The concept of conversion surgery in the management of CCA, particularly in patients with tumours deemed borderline resectable, is gaining significant traction. This strategy involves using neoadjuvant therapies to downstage the tumour and facilitate surgical resection, which remains central to potentially curative treatment of CCA. Recent advances, including the use of HAIC, combined with systemic chemotherapy studies^225^ have shed light on the promising impact of neoadjuvant chemotherapy regimens on the feasibility of resection and subsequent patient outcomes, marking a pivotal shift in the therapeutic landscape of this challenging malignancy. In the study of Matsuyama *et al*.^226^, patients with borderline resectable pCCA received a combination of gemcitabine and S-1 as neoadjuvant chemotherapy. The regimen showed a significant disease control rate of 91.3%, with 71% of patients proceeding to resection. Impressively, 81% of these resected patients achieved an R0 resection^226^. This approach not only increased the resectability rate but also translated into a marked improvement in survival, with a median survival time of 50.1 months in resected patients, compared with 14.8 months in unresected patients^226^. Similarly, Edeline *et al*.^224^ explored the efficacy of combining selective internal RT with chemotherapy in patients with unresectable iCCA. In that study, the response rate was 39% at 3 months, with 22% of patients being downstaged to surgical intervention^224^. This multimodal approach yielded a median overall survival of 22 months^224^, which that surpassed historical outcomes achieved with chemotherapy alone. A recent multicentre study^227^ conducted by the Japanese Society of Hepato-Biliary-Pancreatic Surgery and the Korean Association of Hepato-Biliary-Pancreatic Surgery included 56 patients with initially unresectable locally advanced BTC. Of these patients, 55 (98.2%) received chemotherapy and 16 (28.6%) received additional radiation therapy^227^. The chemotherapy regimens primarily included gemcitabine and cisplatin, with some patients receiving additional agents such as S-1. The median time from the start of initial treatment to resection was 6.4 months. Severe postoperative complications (Clavien–Dindo grade III or higher) occurred in 34 patients (60.7%), postoperative mortality was observed in 5 patients (8.9%), and postoperative histological results revealed a complete response in 8 patients (14.3%). The median survival time from the start of the initial treatment for all patients who underwent conversion surgery was 37.7 months, with 3-year and 5-year survival rates of 53.9 and 39.1%, respectively^227^ .

A recent systematic review and meta-analysis^228^ aimed to evaluate the survival benefit of conversion surgery for initially unresectable BTC. A total of 96 observational studies involving 466 patients were included, with a distribution of 49.6% of patients with iCCA, 28.1% of patients with extra-hepatic CCA, and 22.3% of patients with gallbladder cancer. Of these patients, 52.3% were unresectable due to locally advanced disease, 38.9% were unresectable due to metastatic disease, and 8.7% were unresectable due to a combination of the two. The 90-day mortality rate was 4.3% and the median survival duration was 36.8 months. Overall survival rates at 1, 3, and 5 years were 86%, 59.9%, and 41.1%, respectively^228^. The meta-analysis indicated that patients who underwent conversion surgery had significantly higher survival rates than those who received non-surgical treatment (HR 0.39; *P* < 0.001) and similar survival rates to those who underwent up-front surgery for resectable cancer (HR 1.02; *P* = 0.43)^228^. In addition, male sex and lymph node metastasis were independently associated with reduced overall survival, whereas the presence of metastatic disease did not affect survival. The study^228^ concluded that conversion surgery offers favourable long-term survival and may be a promising option for the multimodality treatment of primary unresectable BTC.

In the NEO-GAP trial^229^, Maithel *et al*. investigated the feasibility of neoadjuvant gemcitabine, cisplatin, and nab-paclitaxel for resectable high-risk iCCA. Despite a notable incidence of grade ≥3 treatment-related adverse events, 73% of patients completed both chemotherapy and surgery. The disease control rate stood at 90%, highlighting the regimen's effectiveness in tumour management. Notably, the completion of neoadjuvant therapy and subsequent surgical resection did not compromise perioperative outcomes, with a median hospital stay of just 4 days. Moreover, the approach facilitated a median overall survival of 24 months across the cohort, with survival not yet reached during follow-up time in those who underwent resection^229^. The inspiration for that neoadjuvant trial was a phase II trial in which 60 patients with advanced unresectable CCA were treated with the same regimen^230^. Notably, 20% of patients were converted to resectability and went on to receive conversion surgery, which is consistent with early outcomes^230^, highlighting the efficacy of this chemotherapy regimen. Collectively, these studies demonstrate the transformative potential of combinatorial neoadjuvant therapies in converting borderline or initially unresectable CCA to resectable CCA, thereby opening the door to curative surgery.

The impact of such therapies extends beyond mere tumour shrinkage, affecting surgical outcomes, lymphadenectomy results, and, critically, patient survival. Neoadjuvant regimens have been shown to significantly improve resection rates, achieve higher rates of R0 resection, and potentially alter the lymph node involvement status, which are key prognostic factors in CCA. Consequently, conversion surgery, facilitated by effective neoadjuvant treatments, represents a beacon of hope for patients with CCA, offering a chance for prolonged survival and potentially curative outcomes in a landscape where treatment options were previously limited. At the same time, it is important to realize that conversion surgery benefits from the ultimate patient selection tool: an *in situ* test of tumour biology and therapeutic efficacy in a patient fit enough to tolerate therapy and be considered for surgery. In this sense, conversion surgery represents the natural progression of the treatment approach to initially unresectable tumours.

### Liver transplantation

In recent years, LT has gained significant traction as a therapeutic option for patients with CCA, particularly when the tumour is deemed unresectable due to advanced local growth or unfavourable anatomical location. Although, historically, LT for CCA was associated with poor outcomes due to the high recurrence rates and postoperative complications, a paradigm shift occurred with the advent of neoadjuvant chemoradiation protocols^231^. These protocols, especially the Mayo Clinic protocol for pCCA, have demonstrated impressive outcomes by stringently selecting candidates and applying multimodal preoperative treatments resulting in substantial increases in overall survival and recurrence-free survival. Post-LT 5-year overall survival rates of over 50%, and in some cases up to 82%, have been reported^232^, which were previously unattainable with resection alone. These improved survival rates align with outcomes of LT for HCC under the Milan criteria, suggesting that LT could be expanded as a standard treatment modality for CCA. In the case of iCCA, where LT was traditionally contraindicated, emerging evidence suggests potential for transplantation in carefully selected patients, especially with early-stage tumours ≤ 2 cm, which show promising outcomes similar to pCCA when coupled with neoadjuvant therapies. Although the data are limited and heterogeneous, these findings open avenues for LT in a subgroup of patients who previously had very limited treatment options.

The meticulous selection of patients based on tumour size and the absence of metastasis, coupled with rigorous neoadjuvant protocols, allows for a reduction in tumour bulk. This downsizing increases the likelihood of achieving negative margins during transplantation and provides a more favourable scenario for complete lymphadenectomy, a critical factor in CCA given its pattern of nodal spread^233^. In addition, transplantation offers a unique advantage over resection in pCCA by removing the entire at-risk extrapancreatic biliary tree, thereby eliminating the possibility of skip lesions and residual microscopic disease that could precipitate recurrence. The adoption of LT for CCA is supported by improvements in neoadjuvant therapies and better understanding of tumour biology. Future research, including prospective multicentre studies, will be necessary to refine the criteria, optimize neoadjuvant protocols, and solidify the role of LT in the management of CCA, potentially shifting the paradigm of CCA treatment towards LT for both pCCA and select patients with iCCA.

## Take-home messages and future perspectives

### Oesophageal cancer

The absence of dedicated data on the incidence, outcomes, and management of induced OMD in oesophageal cancer underscores the critical need for new prospective studies tailored specifically to this patient population. These studies should prioritize patient selection criteria and the development of tools to identify suitable candidates for local treatment of oligometastases, such as validating the utility of ctDNA in this context. In addition, addressing the importance of PET/CT and MRI on indication with the marking of the metastatic site before induction chemotherapy is paramount. Developing criteria to guide the selection of lesions and primary tumour for treatment represents another pivotal aspect to be explored in future research. By focusing on these areas, future studies can enhance understanding and optimize the management of induced OMD in oesophageal cancer.

Specific data regarding the incidence and outcomes following conversion surgery for induced OMD in oesophageal cancer are currently lacking. Findings from a disease-agnostic trial suggest that despite successful systemic therapy leading to the transition from polymetastatic to OMD, the induced OMD state may still exhibit unfavourable biological characteristics compared with other genuine OMD subtypes. These results suggest that selection criteria for the local treatment of induced OMD should be stricter than for other types of OMD.

### Gastric cancer

The RENAISSANCE trial^14^ and the SURGIGAST trial^234^ are both ongoing phase III RCTs evaluating conversion surgery for stage IV gastric cancer. The PERISCOPE II trial^235^ is a phase III RCT evaluating cytoreductive surgery/HIPEC *versus* systemic treatment for gastric cancer-associated peritoneal carcinomatosis and/or positive cytology. Moreover, PIPAC, a novel minimally invasive technique, is currently being investigated in the phase III RCT PIPAC VEROne^236^ for its ability to enhance the possibility of undergoing cytoreductive surgery/HIPEC for gastric cancer-associated peritoneal carcinomatosis and/or positive cytology. Several new important RCTs are planned in metastatic gastric cancer patients, including two pragmatic phase III trials: CONVERGENCE, which aims to compare conversion surgery to continued systemic therapy in patients with limited peritoneal metastases; and IPa-Gastric, the first Western trial addressing IPC in gastric cancer with peritoneal disease.

### Pancreatic cancer

Neoadjuvant therapy is increasingly being administered to patients with all stages of PDAC. Multiagent chemotherapy and/or chemoradiation may facilitate the resection of as many as one-third of patients with LAPC and may be used to select patients with oligometastatic PDAC for subsequent pancreatectomy. Currently, there are several ongoing clinical trials investigating the role of conversion surgery for oligometastatic pancreatic cancer following systemic chemotherapy. The HOLIPANC trial^237^, for example, will include patients with oligometastatic PDAC with a maximum of five metastases limited to the liver, assessing the efficacy and safety of neoadjuvant chemotherapy with liposomal irinotecan combined with oxaliplatin and 5-fluorouracil/folinic acid, followed by curative surgical resection. Another study, the METAPANC trial^238^, is a phase III RCT comparing multimodal therapy, including perioperative modified FFX and surgical resection of both the primary tumour and metastases, to modified FFX chemotherapy alone. The aim of the METAPANC trial is to evaluate overall survival, progression-free survival, and quality of life among patients with resectable PDAC and limited (three or fewer) liver metastases. The same definition for oligometastatic PDAC (three or fewer liver metastases) is being used in the SONAR trial (NCT06690528), which is already currently recruiting patients with response or stability after chemotherapy and randomizing them to either surgery or standard of care. The results of these trials, and others, will be informative as to the feasibility of surgical resection of the primary and hepatic OMD and its effect on overall survival compared with a historical control cohort of those treated with systemic therapy alone.

With regard to conversion surgery for LAPC in select patients, future research efforts should focus on patient selection, standardization of practice, and the quality of life of resected patients, which is usually neglected but should be an important metric together with overall survival. All such patients should be cared for at experienced high-volume centres because a multidisciplinary approach is needed for optimal outcomes. A notable example of this concept is the PREOPANC-4 (NCT05524090), a non-RCT that is enrolling patients in the Netherlands, wherein the international best-practice for LAPC care is implemented nationwide in close collaboration with four international expert centres and proctors.

Surgical expertise is also typically required for vascular reconstructions and high-level postoperative care. Significant improvements in the treatment of pancreatic cancer have been seen in the past decade, with treatment of LAPC and OMD being prime examples. Additional biomarkers, diagnostics, and therapies are an ongoing need for pancreatic cancer in the age of precision medicine and individualized approaches to management.

### Hepatocellular carcinoma

Ongoing clinical trials are exploring various combination regimens to optimize conversion therapy for HCC. A Japanese trial^239^, for example, is assessing the efficacy of lenvatinib followed by conversion surgery in patients with initially unresectable HCC (jRCTs031190057). Future perspectives include refining patient selection criteria, optimizing treatment protocols, and determining the ideal timing for surgery to maximize outcomes. The integration of precision medicine approaches and the development of novel biomarkers to predict response to conversion therapy are also anticipated to enhance the success rates of these interventions. The complexity of HCC management (that is, the prognostic impact of underlying liver disease and patient frailty, the availability of many effective therapeutic options) makes it extremely difficult to find a shared and comprehensive definition of conversion therapy in HCC. Moreover, there is considerable heterogeneity in the definitions of unresectable, unsuitable for ablation, and unsuitable for transplantation, which introduces a large grey area of potential overlap between the concepts of conversion and neoadjuvant therapies. There is an urgent need for the surgical community to make a common effort to find unambiguous and widely shared definitions.

### Colorectal liver metastases

The complexity of conversion therapy mandates management within a multidisciplinary team with access and knowledge of all available treatment modalities. Improved target accuracy and ablation volume prediction and verification will reduce the local recurrence rate and increase the use of thermal ablation as the sole treatment or in combination with resection for otherwise unresectable CRLM. The impressive findings from the TransMet trial^201^, demonstrating an HR of 0.16 for overall survival with chemotherapy and transplantation *versus* chemotherapy alone in highly selected patients with truly unresectable CRLM, suggest an impending expansion in the field of transplant oncology. This may involve investigating the potential of transplantation in patients in need of conversion therapy. Information from liquid biopsies and tissue profiling, combined with the already known clinical biomarkers, will improve patient selection, determining which patients benefit the most from conversion treatment. The ORCHESTRA randomized trial^240^ failed to show a survival benefit of tumour debulking in patients with extensive multiorgan metastases. However, the role of local therapies in the context of liver metastases with unresectable extrahepatic metastases needs to be further explored.

### Biliary tract cancer

As the complexities of BTC are navigated, the imminent future of treatment lies in the realm of precision medicine, propelled by the discovery and integration of robust biomarkers. The nuanced interplay between genetic alterations and the tumour microenvironment presents an opportunity to refine therapeutic approaches for BTC. Advanced genomic profiling has unveiled targets and prompted the development of tailored therapeutics that promise to revolutionize treatment paradigms. The burgeoning field of immunotherapy, particularly with checkpoint inhibitors^241^, is poised to redefine standards of care, although its success hinges on the heterogeneity of individual immune landscapes. Its therapeutic efficacy could be significantly enhanced by gaining ways to modulate the tumour immune microenvironment and deploying combination strategies, marrying immune modulation with chemotherapy or targeted therapy. This multimodal approach could potentially dismantle the barriers erected by the immunosuppressive microenvironment typical of BTC. A crucial advance in the treatment landscape will be the development of non-invasive liquid biopsies for real-time monitoring of disease progression and therapy resistance^242^. As a more granular understanding of resistance mechanisms is gained, including drug efflux modulation, enhanced DNA repair, and survival pathway activation, these biopsies could facilitate dynamic treatment adjustments tailored to the tumour's current state, improving outcomes. Moreover, artificial intelligence and evolving computational methods may soon offer predictive analytics for patient outcomes and guide the selection of combination therapies^243^. Research into patient-derived organoids also holds promise, with the organoids potentially serving as personalized avatars to test the efficacy of drugs before their administration to patients. Finally, prospective trials that are underway will help clarify the role of LT for iCCA (LIRICA; NCT06098547) and further develop the already existing indications for LT in pCCA (LITALHICA; NCT06125769).

## Conclusion

Despite the considerable heterogeneity of its possible targets, few supported by high-quality evidence, and the absence of an accepted standard definition, conversion surgery seems a powerful strategy in the surgical oncologist's toolbox to prolong the overall survival of patients with unresectable UGI and HPB malignancies. Continuous improvements in non-surgical therapies to control the systemic burden and local extension of cancer diseases, supported by improved surgical outcomes for advanced resections in expert centres, explains the increasing interest of the surgical scientific community in the concept of conversion surgery. However, most of the evidence on conversion surgery for UGI and HPB malignancies remains low level and at very high risk of selection bias. In the effort to push forward the boundaries of conversion surgery, two crucial key points are needed: (1) a profound understanding of (and respect for) cancer's biology, which remains key to the stratification and selection of appropriate patients to avoid non-therapeutic surgery; and (2) a general, commonly accepted definition of conversion surgery in order to standardize practice, monitor outcomes, and improve the quality of research.

## Data Availability

Not applicable.
