# Correction to: The evolving concept of conversion surgery for upfront unresectable upper gastrointestinal and hepato-pancreato-biliary cancers: comprehensive review

**DOI:** 10.1093/bjsopen/zrag008

**Published:** 2026-04-03

This is a correction to: Giampaolo Perri, Jennie Engstrand, Robin D Wright, Sebastiaan F C Bronzwaer, Tiuri E Kroese, Biying Huang, Belkacem Acidi, Alessandro Vitale, Hop S Tran Cao, Richard van Hillegersberg, Magnus Nilsson, Ernesto Sparrelid, Matthew H G Katz, Giovanni Marchegiani, Umberto Cillo, The evolving concept of conversion surgery for upfront unresectable upper gastrointestinal and hepato-pancreato-biliary cancers: comprehensive review, *BJS Open*, Volume 9, Issue 4, August 2025, zraf070, https://doi.org/10.1093/bjsopen/zraf070

The following change have been made to the originally published manuscript.

The **Acknowledgments** section has been emended to read: “The authors wish to express their appreciation to Professor van Laarhoven and Dr van Rossum for their substantial contributions to the development of the esophageal cancer chapter. Their expertise, critical analysis, and direct input played an integral role in shaping both the content and clinical relevance of the work.” instead of: “None.”

